# White blood cell count and new-onset atrial fibrillation after cardiac surgery

**DOI:** 10.1186/cc13373

**Published:** 2014-03-17

**Authors:** S Dieleman, K Jacob, H Nathoe, M Ten Berg, D Van Osch, J Frencken, D Van Dijk

**Affiliations:** 1 Utrecht University Medical Center, Utrecht, the Netherlands Critical Care

## Introduction

Postoperative new-onset atrial fibrillation (PNAF) is the most common complication after cardiac surgery. Inflammation as an underlying mechanism has been studied by various inflammatory markers, and white blood cell count (WBC) is the only present consequent inflammatory marker predicting PNAF [1]. This study aimed to determine the association between perioperative WBC and PNAF.

## Methods

Patients >18 years undergoing elective cardiac surgery with a sinus rhythm preoperatively were recruited from the Dexamethasone for Cardiac Surgery-PNAF trial for this *post-hoc *cohort study. The WBC was prospectively measured preoperatively and once during each of the first four postoperative days. Development of PNAF was evaluated with continuous 12-lead ECG monitoring the first 5 days postoperatively.

## Results

**Figure 1:**
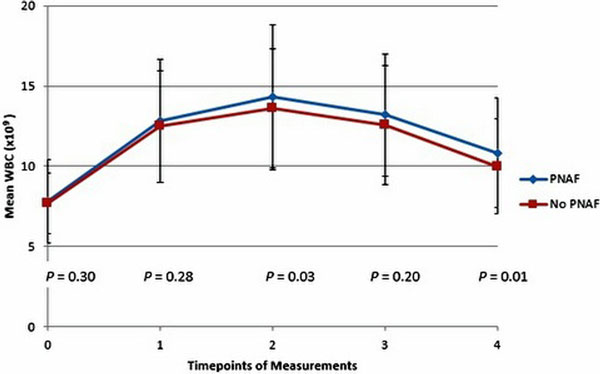
**WBC at baseline *(t *= 0) and in the four postoperative days *(t *= 1 to 4), PNAF versus no PNAF**.

A total of 657 patients were included in this trial, 277 developed PNAF. The WBC was significantly higher in the PNAF group on day 2 and day 4 (Figure 1). However, multivariate analysis showed that preoperative and postoperative WBC, days 1 to 3, were not associated with PNAF (Table 1). Older age (OR: 1.05; CI: 1.03 to 1.07; *P *< 0.001), CABG plus valve surgery (OR: 2.95; CI: 1.78 to 4.88), single valve surgery (OR: 3.09; CI: 2.03 to 4.69; *P *< 0.001) and other surgery (OR: 2.21; CI: 1.23 to 3.97; *P *< 0.001) were correlated with the occurrence of PNAF.

**Table 1:** Multiple regression analysis of association between high WBC and developing PNAF

Time point	OR	95% CI
Baseline	1.04	0.96 to 1.13
Day 1	1.03	0.98 to 1.08
Day 2	1.03	0.99 to 1.08
Day 3	1.03	0.96 to 1.11
Day 4	1.09	1.01 to 1.16

## Conclusion

Preoperative and postoperative WBC were not associated with development of PNAF.
